# Haploid induction via unpollinated ovule culture in *Gerbera hybrida*

**DOI:** 10.1038/s41598-020-58552-z

**Published:** 2020-02-03

**Authors:** Fan Li, Ying Cheng, Xiaokun Zhao, Rongpei Yu, Huimin Li, Lihua Wang, Shenchong Li, Qinli Shan

**Affiliations:** 1 Floriculture Research Institute, Yunnan Academy of Agricultural Sciences, National Engineering Research Center for Ornamental Horticulture, Key Laboratory for Flower Breeding of Yunnan Province, Kunming, 650200 China; 2 School of Agriculture, Yunnan University, Kunming, 650504 China

**Keywords:** Plant breeding, Plant regeneration

## Abstract

Ovule-derived haploid culture is an effective and important method for genetic study and plant breeding. *Gerbera hybrida* is a highly heterozygous species, and the lack of homozygous lines presents a challenge for molecular genetic research. Therefore, we performed haploid induction through unpollinated ovule culture and evaluated the effects of several important factors on this culturing procedure in *G. hybrida*, including genotype, low temperature, and the development seasons of the ovules. Among 45 *G. hybrida* cultivars analyzed, 29 cultivars exhibited adventitious bud induction via *in vitro* unpollinated ovule culture with significant different responses, indicating that the genotype of donor plants was a vital factor for inducibility. Four cultivars with significantly different induction rates, including one non-induced cultivar, were selected to analyze seasonal effects. Ovules extracted in the summer consistently had the highest induction rates, and even the non-induced cultivar included in the analysis could be induced at low levels when ovules from summer were used. Low temperature treatment could also promote adventitious bud induction, and in particular, a strong and significant effect was detected after 7 days of cold treatment. Ploidy level measurements by flow cytometry revealed that 288 ovule-derived regenerants were haploid (55.17%) and 218 lines were diploid (41.76%). Moreover, genetic stability analysis of the regenerants indicated 100% similarity to the marker profile of the mother plant. This is the first report of ovule-derived haploids in *G. hybrida*, which may facilitate the development of homozygous lines for molecular research and plant breeding.

## Introduction

*Gerbera hybrida* is one of the most popular ornamental plants worldwide^1^. Since most of the commecial cultivars were bred from the crossing of wild-type *G. jamesonii* and *G. viridifolia*, the genome of *G. hybrida* is highly heterozygous^2^. Hundreds of *G. hybrida* varieties with extremely rich flower color patterns are available in the flower market, including white, yellow, red, pink, purple, and brown, and *G. hybrida* ranks fourth in cut flowers after rose, chrysanthemum, and tulip^3^. As a highly heterozygous species, *G. hybrida* naturally harbors genetic diversity, which is beneficial for hybridization breeding. However, the lack of a homozygous genome is challenging for molecular genetic research, including forward genetic screening and mutation mapping.

Ploidy breeding, the modification of the number of chromosome sets in a plant genome, is frequently used in ornamental plant breeding to induce novel variation and to create homogenous lines^4^. A commonly used protocol for ploidy breeding is haploid induction, which refers to haploid regeneration via a single gamete cell under specific conditions, using either pollen (anther or microspore culture) or egg cells (ovule culture)^5^. Essentially, the applicability of haploid induction is the main trigger for ornamental breeding, and some haploids of ornamentals have been commercially developed, including the horticultural variety *Pelargonium* ‘Kleine Liebling’^6^. Nevertheless, haploid induction, particularly induction via ovule culture, has only been reported in a limited number of ornamental species. An extensive review of haploidization in ornamental species listed 46 ornamental genera in which haploidization had been accomplished through one or multiple techniques, and ovule/ovary culture was reported in only six genera^7^. Moreover, the haploidization protocols are highly species specific, with genotype as a crucial factor for successful haploidization within a species^8^. In the Asteraceae family, different *in vitro* culture methods for haploid induction have been reported, such as haploidization from unfertilized ovules in *G. jamesonii*^9–11^ and *Chrysanthemum morifolium*^12^. However, similar strategies have not yet been reported in the most relevant ornamental species *G. hybrida*, even though this species has been used as an Asteraceae model plant for decades.

In this study, we conducted *in vitro* unpollinated ovule culture to induce haploidization in *G. hybrida* and evaluated the effects of the following factors: genotype, low temperature, and the development seasons of the ovules. First, we analyzed 45 *G. hybrida* commercial cultivars to measure the rate of adventitious bud induction and to assess the response to unpollinated ovule culture. Four *G. hybrida* cultivars with significantly different induction rates were selected, and for these cultivars, ovules from different seasons and 4 °C cold treatment were applied to optimize the conditions and ovule explants for unpollinated ovule culture. The eventual goal was to create regenerated haploids, which were confirmed by flow cytometry measurements. Our study provides a basic strategy for haploid induction via unpollinated ovule culture in *G. hybrida*, which may greatly facilitate the generation of homozygous lines for molecular research as well as doubled haploid lines for plant breeding.

## Results

### Analysis of unpollinated ovule culture in 45 cultivars

**Figure 1:**
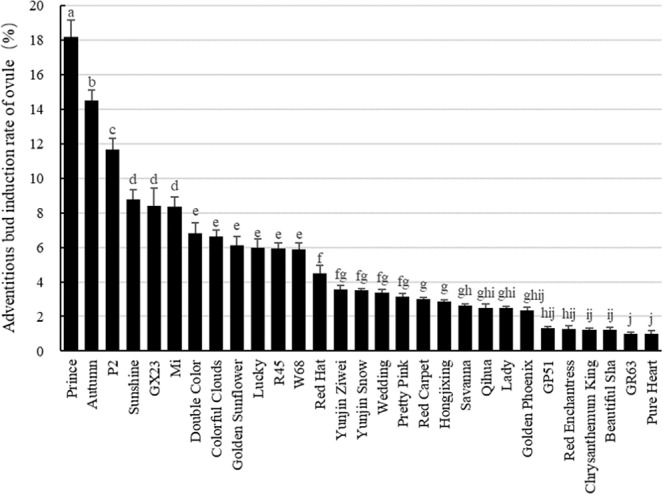
Adventitious bud induction rate of ovules from different *Gerbera hybrida* cultivars. One-way ANOVA was used to assess statistical significance, and *p* values were calculated with Tukey’s HSD test (α = 0.05). The detailed data for this figure are available in Table S1.

First, we investigated the effect of genotype on unpollinated ovule culture. For this analysis, 45 different cultivars were used to obtain ovule explants from semi-open flowers. The unpollinated ovules were then cultured *in vitro* to determine whether adventitious buds could be induced. The results showed that 29 cultivars exhibited adventitious bud induction through unpollinated ovule culture, accounting for 64.44% of all tested varieties (Fig. 1). The rate of adventitious bud induction was over 5% in 12 cultivars, and the highest induction rate was detected in cultivar ‘Prince’ (18.19% ± 0.96, n = 3). These experimental data indicate that the ovules from different cultivars respond differently to unpollinated ovule culture, reflecting differences in their genetic backgrounds and genotypes, as each variety was derived from different hybrid lines.

**Figure 2:**
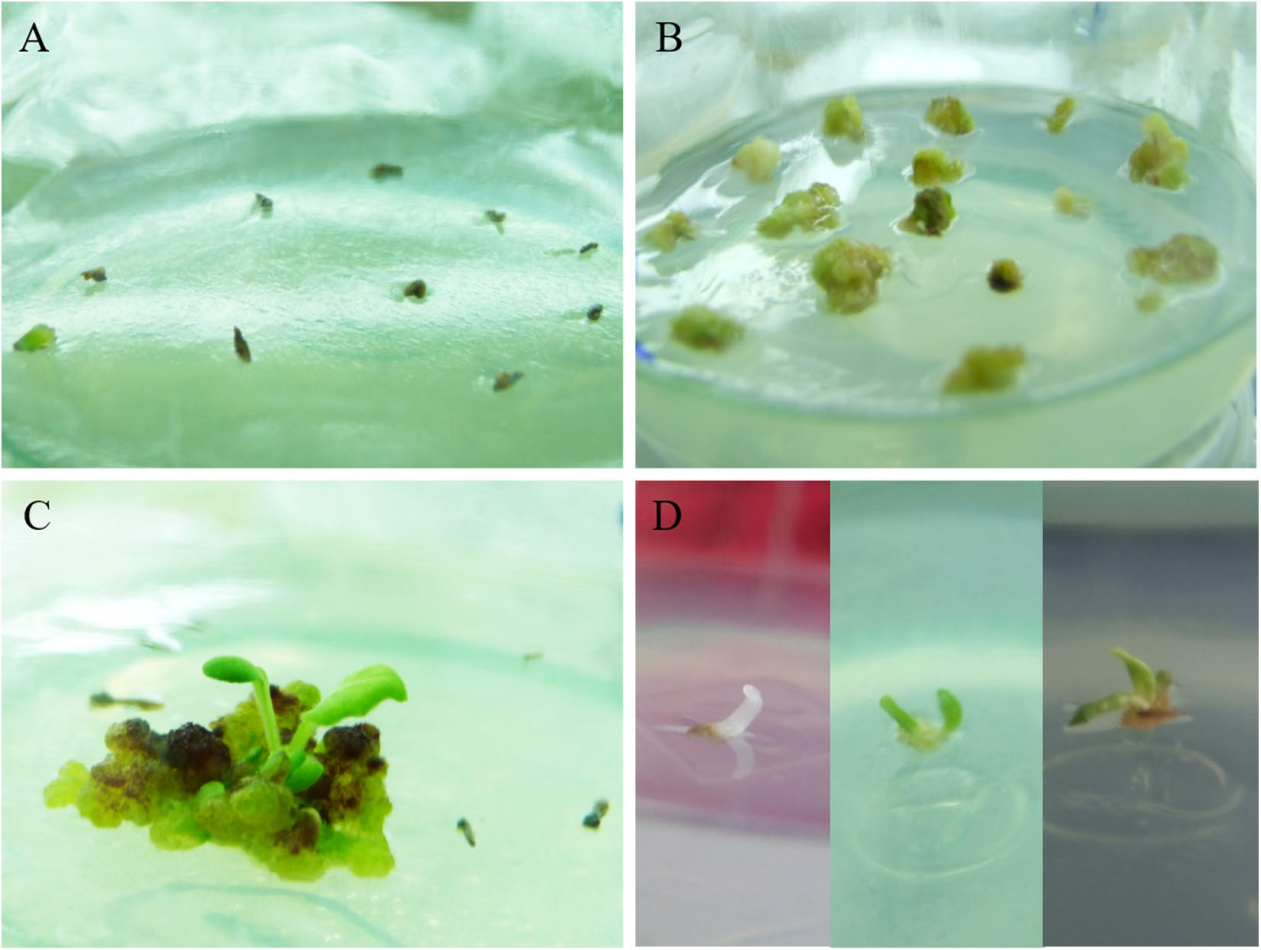
The stages of ovule induce to form adventitious buds in *Gerbera hybrida*. (**A**) Gradual expansion of the ovule volume; (**B**) callus formation; (**C**) adventitious bud induction. (**D**) In some cases, an adventitious bud formed directly from an induced ovule without callus formation, and three distinguishable stages were displayed.

During the process of ovule induction, three distinct stages were identified from ovules to regenerants: (1) gradual expansion of the ovule, (2) callus formation, and (3) adventitious bud induction (Fig. 2). However, adventitious bud induction was not always preceded by callus formation, and we observed a small number of ovules that were directly induced to form adventitious buds (Fig. 2). When adventitious buds formed in this manner, the induction time was shorter than that of buds formed via callus formation.

### The effect different of seasons on unpollinated ovule culture

Based on the above analysis of ovule induction in 45 different cultivars of *G. hybrida*, four cultivars with significantly different adventitious bud induction rates (‘Prince’, ‘Sunshine’, and ‘Hongjixing’ along with the non-induced cultivar ‘Dolly’) were selected for further study. Ovule explants were obtained from different seasons (spring, summer, autumn, and winter) and used for unpollinated ovule culture. Our results indicated that ovule explants from summer had the highest adventitious bud induction rates and the shortest induction times (Table 1). Although the cultivar ‘Dolly’ was not induced in the preliminary analysis, adventitious buds were induced at a very low rate in this cultivar when ovules from spring and summer were used. Generally, there were no significant differences in the induction effect between ovules obtained in spring and autumn, but ovules obtained in winter had the lowest induction rate, indicating that temperature may also significantly influence unpollinated ovule culture.

**Table 1:** Seasonal effects on unpollinated ovule culture in *Gerbera hybrida*.

Cultivar	Season	Adventitious bud induction rate (%)	Adventitious bud induction time (Days)
‘Prince’	Spring	18.33 ± 1.44^ab^	45.33 ± 0.58^l^
	Summer	20.00 ± 2.50^a^	39.33 ± 1.53^m^
	Autumn	17.50 ± 2.50^ab^	48.33 ± 0.58^k^
	Winter	15.83 ± 1.44^bc^	51.00 ± 1.00^j^
‘Sunshine’	Spring	12.50 ± 2.50^d^	76.67 ± 0.58^e^
	Summer	13.33 ± 1.44^cd^	73.33 ± 1.53^f^
	Autumn	11.67 ± 1.44^de^	79.00 ± 1.00^d^
	Winter	9.17 ± 1.44^ef^	82.33 ± 1.53^c^
‘Hongjixing’	Spring	5.83 ± 1.44^gh^	70.00 ± 1.00^h^
	Summer	8.33 ± 1.44^fg^	60.33 ± 1.53^i^
	Autumn	4.17 ± 1.44^hi^	71.00 ± 1.00^gh^
	Winter	1.67 ± 1.44^ij^	72.00 ± 1.00^fg^
‘Dolly’	Spring	0.83 ± 1.44^j^	89.00 ± 1.00^a^
	Summer	1.67 ± 1.44^ij^	87.00 ± 2.00^b^
	Autumn	\	\
	Winter	\	\

One-way ANOVA was used to assess statistical significance, and *p* values were calculated with Tukey’s HSD test (α = 0.05). The data indicate the mean value ± standard deviation from three replicates. The detailed data for this table are provided in Table S2.

Among the four cultivars, ‘Prince’ had the highest induction rate and the shortest induction time in summer, suggesting that it may be a suitable material for further analysis of *G. hybrida* unpollinated ovule culture. In contrast, it was difficult to induce adventitious buds in the cultivar ‘Dolly’, underscoring the impact of genotype on the induction of *G. hybrida* through unpollinated ovule culture.

### The effect of low temperature on unpollinated ovule culture

Next, we investigated the effect of low temperature treatment on the same four cultivars using ovules extracted during the summer. Differences in the duration of low temperature treatment affected the response to unpollinated ovule culture, and for all cultivars, an optimal induction effect was observed with 7 days of treatment (Table 2). Specifically, ‘Prince’ had the highest induction rate (25.79% ± 1.44%, n = 3), which was approximately 5% greater than the induction rate of the control after 7 days of low temperature treatment. Consistent with the previous results, induction of ‘Dolly’ through unpollinated ovule culture was extremely limited, with some adventitious bud formation under low temperature treatment for 3 days and 7 days. Our results show that low temperature treatment could promote the induction of adventitious buds in *G. hybrida*, and the optimal treatment duration was 7 days.

**Table 2:** The effect of low temperature (4 °C) on unpollinated ovule culture in *Gerbera hybrida*.

Cultivar	Treatment time (Days)	Adventitious bud induction rate (%)	Adventitious bud induction time (Days)
‘Prince’	0	20.83 ± 1.44^b^	38.67 ± 0.58^gh^
	3	22.5 ± 2.50^ab^	40.00 ± 1.00^g^
	7	25.83 ± 1.44^a^	37.33 ± 0.58^hi^
	10	24.17 ± 2.89^ab^	36.67 ± 0.58^i^
‘Sunshine’	0	12.50 ± 2.50^de^	69.67 ± 0.58^d^
	3	15.00 ± 2.50^cd^	70.33 ± 0.58^d^
	7	17.50 ± 2.50^c^	72.67 ± 0.58^c^
	10	13.33 ± 1.44^d^	74.00 ± 1.00^c^
‘Hongjixing’	0	8.33 ± 1.44^f^	63.00 ± 1.00^e^
	3	9.17 ± 1.44^ef^	62.33 ± 1.15^e^
	7	12.50 ± 2.50^de^	58.00 ± 1.00^f^
	10	9.17 ± 1.44^ef^	59.00 ± 1.00^f^
‘Dolly’	0	\	\
	3	1.67 ± 1.44^g^	85.33 ± 0.58^b^
	7	1.67 ± 1.44^g^	88.33 ± 0.58^a^
	10	\	\

One-way ANOVA was used to assess statistical significance, and *p* values were calculated with Tukey’s HSD test (α = 0.05). The data represent the mean value ± standard deviation from three replicates. The detailed data for this table are provided in Table S3.

### Evaluation of regenerants

**Figure 3:**
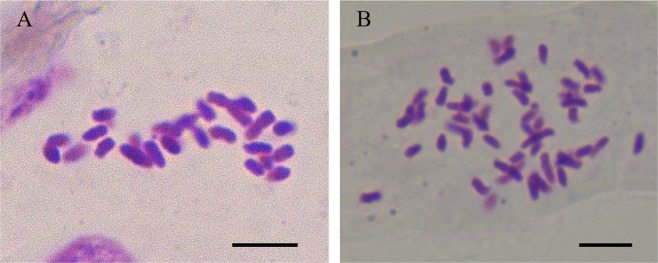
Microscopic view (100 X) of root tip chromosomes of *Gerbera hybrida* ovule-derived haploids and control diploids. (**A**) The chromosomes of ovule-derived haploids (n = 25); (**B**) The chromosomes of control diploids (2n = 50). Bars = 5 μm.

**Figure 4:**
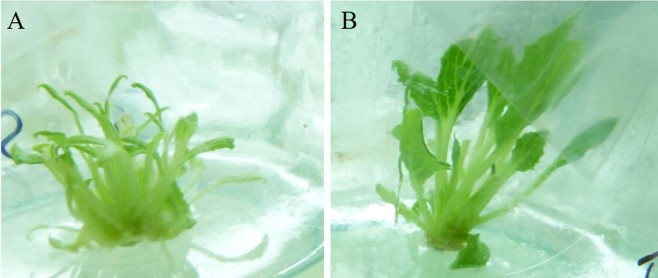
The regenerant phenotype of *Gerbera hybrida* ovule-derived haploids and control diploids. (**A**): The ovule-derived haploids had weak leaves that were short and narrow. (**B**) The control diploid phenotype.

After the induction of adventitious buds, we cultivated each induction line into individual ovule-derived haploid regenerants. First, we examined the chromosome ploidy of ovule-derived haploids and control diploids by root tip chromosome counting. The ovule-derived haploids harboured 25 chromosomes (n = 25, Fig. 3), which was half of the chromosome number of the control diploids (2n = 50, Fig. 3), confirming that the unpollinated ovule culture was successful. Next, using flow cytometry and the leaf materials of ovule-derived haploids and control diploids, we determined the ploidy level of ovule-derived haploids from different diploid cultivars. The results showed that haploids, diploids, and mixoploids co-existed in 522 lines of the ovule-derived regenerated plant population. Specifically, 55.17% were haploids (288 lines), 41.76% were diploids (218 lines), and 16 strains were mixoploids. In terms of plant morphology, the regenerants of the ovule-derived haploids differed markedly from the control diploids and had weak, short, and narrow leaves (Fig. 4). This phenotype is consistent with those of other haploid plants.

True-to-type clonal fidelity is the key prerequisite for *in vitro*-regenerated plantlets, since somaclonal variation may occur in the regenerants. Therefore, we used eight random amplified polymorphic DNA (RAPD) markers to evaluate the genetic stability of the *in vitro*-regenerated plantlets (five individual regenerants) of five *G. hybrida* cultivars (‘Prince’, ‘Sunshine’, ‘Hongjixing’, ‘Dolly’, and ‘Pretty Pink’, Fig. S1). The PCR results showed that all amplified bands from the ovule-derived haploids were monomorphic and similar to those of the mother plant. Moreover, a similarity matrix based on Jaccard’s coefficient showed that the pair-wise value between mother and regenerant plantlets was 1, indicating 100% similarity and no variability among the plantlets derived from unpollinated ovule culture. These findings indicate that ovule explants can be used for *G. hybrida* haploid induction without much risk of genetic instability.

## Discussion

Multiple studies have demonstrated that genotype is a fundamental factor for successful haploid induction via *in vitro* unpollinated ovule culture^8,13,14^. Accordingly, the variety selected as the ovule donor for haploid induction is of utmost importance. The different responses to haploid induction reflect differences between species as well as differences among genotypes of an individual species. For example, genotype proved to be a key factor influencing *in vitro* gynogenesis and androgenesis in summer squash (*Cucurbita pepo*), cucumber (*Cucumis sativus*), winter squash (*Cucurbita maxima*), pumpkin (*Cucurbita moschata*), and watermelon (*Citrullus lanatus*)^13,15–17^. A previous study of *Gerbera jamesonii* including ten different *Gerbera* genotypes showed that genotype affected callus induction, which ranged from 5% to 43%, as well as the shoot regeneration rate, which ranged from 57% to 95%^18^. Several studies have also demonstrated that not all genotypes of *G. jamesonii* can produce haploid callus and shoots. For instance, Tosca *et al*. found that 12 out of 21 genotypes could induce haploid callus and only six could produce shoots^19^. Miyoshi and Asakura reported that 13 out of 17 genotypes could form callus in pot gerbera (*G. jamesonii*)^11^. In line with these previous findings, our analysis of 45 *G. hybrida* cultivars showed that 29 could be induced to form adventitious buds through unpollinated ovule culture, indicating that genotype has a significant effect on unpollinated ovule culture in *G. hybrida*. This difference in induction response may reflect the genetic diversity of the 45 cultivars, as each variety was derived from different genetic backgrounds.

In addition to genotype, other factors that critically affect unpollinated ovule culture include season and temperature. Seasonal effects on ovule culture have been observed in several species, but the available information is limited^8^. Cappadocia *et al*. reported a seasonal effect on *G. jamesonii* callus induction via unfertilized ovules and showed that callus produced in the spring had a higher morphogenetic capacity^20^. Tosca *et al*. also observed seasonal effects on unfertilized ovules in four genotypes of pot gerbera (*G. jamesonii*) from April to October in northern Italy; two genotypes had the highest callus formation in spring, the other two had the highest callus formation in autumn, and gynogenesis did not occur in winter^21^. In the present study, all four cultivars selected for further analysis had the highest rate of adventitious bud induction in summer. Notably, this included the cultivar ‘Dolly’, which did not exhibit adventitious bud induction when using ovules from autumn and winter. These findings indicate that season significantly affects ovule induction in *G. hybrida*, which is consistent with previous reports.

The positive effect of low temperature treatment (4 °C) for 4 to 5 days on *in vitro* androgenesis or gynogenesis has been reported in several species. In ornamental kale (*Brassica oleracea* L. var. *acephala*), cold treatment significantly improved plant regeneration from microspore-derived embryos to a rate of up to 79.0% under 4 °C for 2 days or 5 days^22^. Cold pretreatment (4 °C) of flower buds for 1–2 weeks also promotes effective induction of embryo-like structures in plant regeneration from unfertilized ovule culture of gentians (*Gentiana* spp.)^23^. Cold pretreatment also has a positive effect on direct embryo induction in pepper (*Capsicum annuum* L.)^24^. In our study, 4 °C cold pretreatment of flower buds for 7 days had the strongest effect on unpollinated ovule culture *in vitro* and led to the greatest increase in adventitious bud induction. This finding suggests that low temperature may promote adventitious bud induction in *G. hybrida*, consistent with the findings of previous studies. One possible explanation for this temperature effect is the diverting of normal gametophytic development into a sporophytic pathway, which would therefore promote the formation of haploid embryos^25^.

During the process of generating haploids from cultured ovules, most of the regenerants developed directly from callus, but we also observed a few adventitious buds that skipped the callus formation stage and developed directly from ovule tissues. This phenomenon has been observed previously in other haploid induction studies, and the adventitious buds may arise directly or be produced indirectly through a callus intermediate^26^. Since callus may form from somatic tissue associated with the egg cell, regenerants from callus may be haploid, diploid, or mixoploid^8^. In the present study, 55.17% of the 522 lines of ovule-derived regenerated plantlets were identified as haploid, and 41.76% were diploid. These results are consistent with a previous analysis of unfertilized ovule/ovary culture in gentians (*Gentiana* spp.), with 57.9% haploid and 34.3% diploid among the total of 1,515 regenerated plantlets^23^. Miyoshi and Asakura cultured unpollinated ovules of several genotypes of pot gerbera (*G. jamesonii*) and found that 80.4% were haploid, 15.2% were diploid, and 4.3% were mixoploid^11^. Cappadocia *et al*. similarly obtained 76% haploid plants using unpollinated ovules for *G. jamesonii* haploid production^20^. Among the unpollinated ovule-derived haploids in the present study, no genetic variation was detected by RAPD marker analysis, indicating that *in vitro* unpollinated ovule culture is a reliable method for haploid induction of *G. hybrida* without much risk of genetic instability. This observation is in line with the genetic stability of micropropagated plantlets of *G. jamesonii*, which showed no somaclonal variation during micropropagation^27,28^.

Our study of haploid induction via unpollinated ovule culture in *G. hybrida* demonstrates the applicability and reliability of this technique and provides basic knowledge for haploidization in *G. hybrida*. The findings show that this strategy can be applied to a wide range of *G. hybrida* genotypes. The haploids generated through this approach could potentially be used to create doubled haploids in breeding programs to reduce cultivar development time. The haploid lines could also facilitate molecular research in *G. hybrida*; for example, genome sequencing of haploids would reduce the cost and data volume due to the halved chromosome number.

## Methods

### Plant material and growth conditions

The following 45 commercial cultivars of *Gerbera hybrida* with different flower colors and genetic backgrounds were analyzed in this study: ‘Prince’, ‘Autumn’, ‘Sunshine’, ‘Colorful Clouds’, ‘Double Color’, ‘Golden Sunflower’, ‘Lucky’, ‘Red Hat’, ‘Yunjin Ziwei’, ‘Yunjin Snow’, ‘Wedding’, ‘Pretty Pink’, ‘Red Carpet’, ‘Hongjixing’, ‘Savanna’, ‘Qihua’, ‘Lady’, ‘Golden Phoenix’, ‘Red Enchantress’, ‘Chrysanthemum King’, ‘Beautiful Sha’, ‘Pure Heart’, ‘Flawless Clouds’, ‘Minister’, ‘Big Champagne’, ‘Winter’, ‘Dolly’, ‘Imperial Concubine’, ‘Over Fire’, ‘Red Gorgeous’, ‘Lalissa’, ‘Exquisite’, ‘Rose’, ‘Honey’, ‘Princess’, ‘Cosy Sweet’, ‘Sunshine Coast’, ‘Purple Queen’, ‘P2’, ‘GX23’, ‘Mi’, ‘W68’, ‘R45’, ‘GP51’, and ‘GR63’. The plant seedlings were obtained from Yuxi Yunxing Biological Technology Co., Ltd. (Yunnan Province, China). All seedlings were planted separately in individual pots (diameter = 14 cm, height = 12 cm), which were filled with commercial potting substrate (peat:perlite = 3:1) then cultivated in controlled-environment growth chambers in a lab at the Yunnan Academy of Agricultural Sciences. The conditions were as follows: 25 °C, 12 h photoperiod (240 μmol/m^2^s photosynthetic photon flux density), and 55% relative humidity^3^.

### Selection of ovule explants

**Figure 5:**
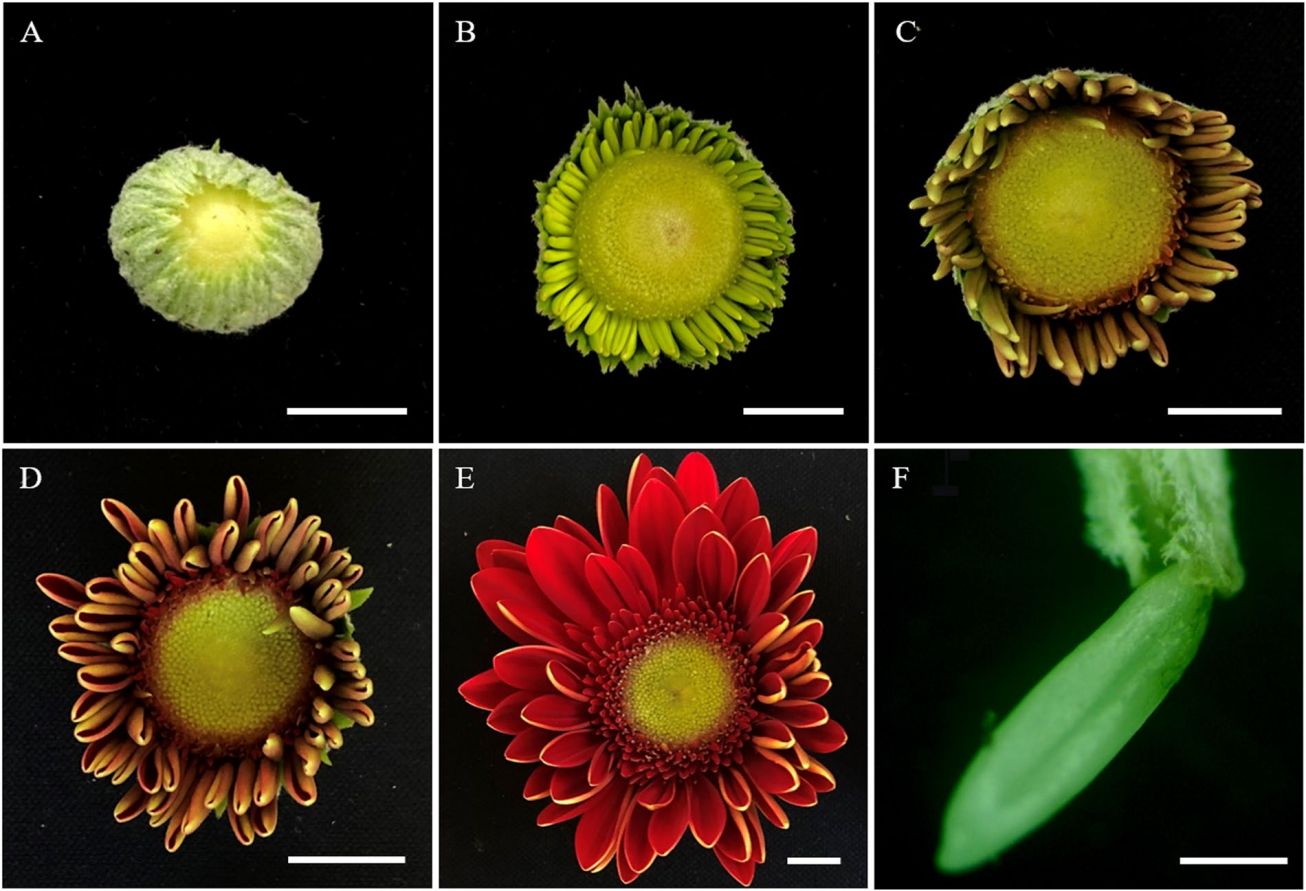
The five flower development stages of *Gerbera hybrida* and an ovule explant from a semi-open stage flower. (**A**) flower bud stage; (**B**) sepal open stage; (**C**) ray florets colored stage; (**D**) ray florets open stage; (**E**) flower semi-open stage. (**F**) Stripped ovule. Bars = 1 cm in (**A–E**) and 500 μm in F.

*Gerbera hybrida* flower development can be divided into five stages: flower buds, sepal open, ray florets colored, ray florets open, and flower semi-open. In this study, the ovule explants were obtained from semi-open stage flowers (Fig. 5). First, the semi-open flowers were washed with detergent and rinsed with deionized water. On an ultra-clean workbench, the ray florets at the edge of the inflorescence were cut and removed using a sharp surgical blade, followed by disinfection with 0.1% HgCl_2_, 2% NaClO, and deionized water for 15 minutes, 10 minutes, and 5 minutes, respectively. Finally, the ovules of disc florets at the center of the inflorescence were dissected and peeled off under a microscope. Ovules with a full appearance and no mechanical damage were used as explants.

### *In vitro* unpollinated ovule culture

The unpollinated ovules were cultured *in vitro* in MS medium (0.6 mg/L BA, 0.1 mg/L NAA, 30 g/L sugar, 7 g/L agar, pH = 5.8) in controlled-environment chambers at 22 °C in the dark for 7 days then transferred to a 10 h photoperiod and 50 μmol/m^2^s photosynthetic photon flux density. The ovules were transferred to fresh medium every 30 days. After 90 days, the adventitious bud induction rate was calculated using the following formula: induction rate (%) = number of ovules with induced adventitious buds/number of cultured ovules. Induction time was quantified as the average time for adventitious bud formation from three replicates.

### Ovule explants from different seasons and low temperature treatment

The *Gerbera hybrida* cultivars ‘Prince’, ‘Sunshine’, ‘Hongjixing’, and ‘Dolly’ had significantly different adventitious bud induction rates and were selected to further study the effect of different seasons on unpollinated ovule culture. Seedlings of these four cultivars were planted in a greenhouse under natural conditions. Semi-open flowers of each cultivar were collected in spring (April), summer (July), autumn (October), and winter (January) to extract the ovules that developed in different seasons. Three individual plants were used as replicates for each treatment.

These four cultivars were also used to investigate the effects of low temperature treatment on unpollinated ovule culture during the summer season. The plants were subjected to low temperature treatment (4 °C) for 0 days, 3 days, 7 days, and 10 days in controlled-environment chambers, followed by ovule extraction. Three individual plants were used as replicates for each treatment.

### Identification of ovule-derived haploid regenerants

The ovule culture-derived haploid plants were identified using a flow cytometer (CyFLOW Cube, Partec GmbH, Germany), equipped with a UV lamp emitting at 358 nm and a TK 420 filter, together with chromosome ploidy analysis software. Young leaf tissue samples (10 cm^2^) from the tested plants were placed in a clean culture dish with 500 μL extraction lysis buffer (HR-A, Partec High Resolution Staining Kit). The leaf tissue was rapidly chopped using a sharp surgical blade then completely immersed in the extraction lysis buffer for 5 minutes in the dark. Next, 1600 μL DAPI staining buffer (HR-B, Partec High Resolution Staining Kit) was added and mixed, followed by a 5 minute incubation in the dark. Lastly, the leaf samples were processed through a filter with a pore size of 20 μm (Partec Cell Trics), and the leaf cell suspensions were transferred into a U-shaped tube for flow cytometry analysis under an excitation wavelength of 350 nm.

**Figure 6:**
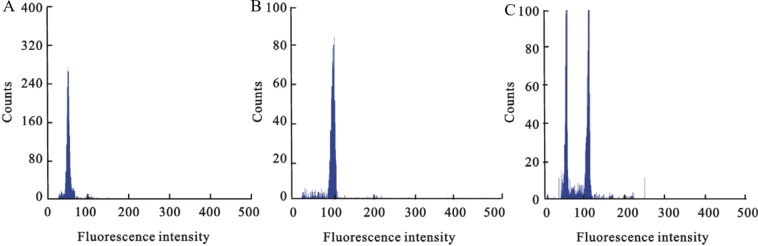
Flow cytometry chromosome fluorescence intensity histograms for different ploidy levels of *Gerbera hybrida*. (**A**) Intensity of haploid *G. hybrida*; (**B**) intensity of diploid *G. hybrida*; (**C**) intensity of mixoploid *G. hybrida*.

The leaves of haploid and diploid plants that had been confirmed by root tip chromosome counting were used as standards for the flow cytometry analysis. Specifically, the fluorescence intensity of the chromosome separation peak was adjusted to 50 for haploid samples; for diploid samples with double the chromosome number, a fluorescence intensity peak occurred at 100 (Fig. 6). Three leaves from each tested plant were measured as three samples, and each plant was measured three times. The fluorescence intensity of the chromosome separation peak was used to distinguish haploid, diploid, and mixoploid.

### Genetic stability evaluation of ovule-derived haploid regenerants

Since the plantlets were derived through callus, somaclonal variation may have occurred. To evaluate the genetic fidelity of the ovule-derived haploid regenerants, eight random amplified polymorphic DNA (RAPD) markers were used to analyze the *in vitro*-regenerated plantlets of five *G. hybrida* cultivars (‘Prince’, ‘Sunshine’, ‘Hongjixing’, ‘Dolly’, and ‘Pretty Pink’). Five ovule-derived haploid regenerants were chosen randomly from the population and compared with the mother plant from which the ovule explants were taken. The genomic DNA of mother plants and regenerants were extracted from young leaves using the CTAB method^29^. The RAPD primers were synthesized by the Beijing Genomics Institute (BGI TECH, Shenzhen, China) (Table 3). The 25 μL PCR reaction volume included the following: 2 μL genomic DNA template, 2.5 μL 10 × buffer, 0.5 μL dNTPs, 1 μL primer, 0.3 μL DNA Taq polymerase, 1.5 μL MgCl2 (2 mM), and 17.2 μL nuclease-free water. PCR amplification was performed on a thermal cycler (RePure-A, Bio-Gener, China) using the following PCR reaction conditions: 5 min at 95 °C; followed by 40 cycles of 60 s at 95 °C, 60 s at the optimal annealing temperature, and 120 s at 72 °C; and a final extension of 5 min at 72 °C. The PCR products were separated by 1% agarose gel electrophoresis and stained with ethidium bromide. Each amplified fragment in the size range of 100 bp to 5 kb was manually scored by the following rules according to Jaccard’s similarity coefficient: the presence or absence of bands in the gel were denoted by ‘1’ and ‘0’, respectively^28^.

**Table 3:** Primer sequences and size range of the amplified fragments generated by random amplified polymorphic DNA (RAPD) primers in *Gerbera hybrid*.

Primer name	Primer sequence (5′-3′)	Size of amplification (bp)
OPA-07	GAAACGGGTG	250–2000
OPA-08	GTGACGTAGG	500–1500
OPA-15	TTCCGAACCC	250–2000
OPA-19	CAAACGTCGG	500–3000
OPC-1	TTCGAGCCAG	500–2000
OPC-5	GATGACCGCC	250–1500
OPC-12	TGTCATCCCC	700–3000
OPE-01	CCAAGGTCCC	250–2000

### Statistical analysis

Data analysis and statistics were performed using Microsoft Excel 2016 and Data Processing System^30^. One-way ANOVA with Tukey’s HSD post-hoc test was used to compare multiple samples at the 5% significance level. The data were obtained from three replicates and presented as mean value ± standard deviation in the tables.

## Supplementary information

Supplementary information.
