# Supplementary material for: Haploid induction via unpollinated ovule culture in Gerbera hybrida
Source: Sci Rep. 2020 Feb 3;10:1702. doi: 10.1038/s41598-020-58552-z (PMC6997385; doi:10.1038/s41598-020-58552-z)
Supplement: Supplementary file 1 — Supplementary information. [file 41598_2020_58552_MOESM1_ESM.pdf]

Haploid induction via unpollinated ovule culture in *Gerbera hybrida*

Fan Li[1], Ying Cheng[2], Xiaokun Zhao[2], Rongpei Yu[1], Huimin Li[1], Lihua Wang[1]*, Shenchong Li[1]*, and Qinli Shan[1]*

1. Floriculture Research Institute, Yunnan Academy of Agricultural Sciences, National Engineering Research Center for Ornamental Horticulture, Key Laboratory for Flower Breeding of Yunnan Province, Kunming 650200, China

2. School of Agriculture, Yunnan University, Kunming 650504, China

*These authors jointly supervised this work.

E-mail: wlh2525@21cn.com, lsc0618@163.com, and shqli2008@126.com

Supplementary Information:

Table S1. Detailed data for adventitious bud induction rates of ovules from different *Gerbera hybrida* cultivars.

No.	Cultivar	Adventitious bud induction rate of ovule (%)					
		Repeat 1	Repeat 2	Repeat 3	Average	SD	Sig. (5%)
1	Prince	19.13	18.22	17.22	18.19	0.96	a
2	Autumn	13.96	14.38	15.18	14.51	0.62	b
3	P2	12.39	11.39	11.19	11.66	0.64	c
4	Sunshine	8.15	9	9.18	8.78	0.55	d
5	GX23	9.42	7.39	8.39	8.40	1.02	d
6	Mi	8.98	8.28	7.88	8.38	0.56	d
8	Double Color	7.36	6.18	6.98	6.84	0.60	e
7	Colorful Clouds	7.02	6.61	6.21	6.61	0.41	e
9	Golden Sunflower	6.32	5.5	6.5	6.11	0.53	e
12	Lucky	6.54	5.51	5.91	5.99	0.52	e
11	R45	6.32	5.81	5.61	5.91	0.37	e
10	W68	5.87	5.48	6.28	5.88	0.40	e
13	Red Hat	4.98	4.37	4.07	4.47	0.46	f
14	Yunjin Ziwei	3.33	3.78	3.63	3.58	0.23	fg
15	Yunjin Snow	3.59	3.39	3.49	3.49	0.10	fg
16	Wedding	3.37	3.57	3.17	3.37	0.20	fg
17	Pretty Pink	3.06	3.36	3.02	3.15	0.19	fg
18	Red Carpet	2.89	2.99	3.09	2.99	0.10	g
19	Hongjixing	2.86	2.76	2.96	2.86	0.10	g
20	Savanna	2.63	2.53	2.73	2.63	0.10	gh
21	Qihua	2.5	2.7	2.3	2.50	0.20	ghi
22	Lady	2.5	2.4	2.6	2.50	0.10	ghi
23	Golden Phoenix	2.33	2.53	2.13	2.33	0.20	ghij
24	GP51	1.33	1.23	1.43	1.33	0.10	hij
25	Red Enchantress	1.28	1.46	1.1	1.28	0.18	hij

26	Chrysanthemum King	1.25	1.2	1.3	1.25	0.05	ij
27	Beautiful Sha	1.25	1.35	1.15	1.25	0.10	ij
28	GR63	1	1.1	0.9	1.00	0.10	j
29	Pure Heart	1	1.2	0.8	1.00	0.20	j
30	Flawless Clouds	0	0	0	0.00	0.00	
31	Minister	0	0	0	0.00	0.00	
32	Big Champagne	0	0	0	0.00	0.00	
33	Winter	0	0	0	0.00	0.00	
34	Dolly	0	0	0	0.00	0.00	
35	Imperial Concubine	0	0	0	0.00	0.00	
36	Over Fire	0	0	0	0.00	0.00	
37	Red Gorgeous	0	0	0	0.00	0.00	
38	Lalissa	0	0	0	0.00	0.00	
39	Exquisite	0	0	0	0.00	0.00	
40	Rose	0	0	0	0.00	0.00	
41	Honey	0	0	0	0.00	0.00	
42	Princess	0	0	0	0.00	0.00	
43	Cosy Sweet	0	0	0	0.00	0.00	
44	Sunshine Coast	0	0	0	0.00	0.00	
45	Purple Queen	0	0	0	0.00	0.00	

Table S2. Detailed data for the seasonal effects on unpollinated ovule culture in *Gerbera hybrida*.

Cultivar	Season	Adventitious bud induction rate (%)						Adventitious bud induction time (Days)					
		Repeat 1	Repeat 2	Repeat 3	Average	SD	Sig. (5%)	Repeat 1	Repeat 2	Repeat 3	Average	SD	Sig. (5%)
‘Prince’	Spring	17.5	20	17.5	18.33	1.44	ab	45	45	46	45.33	0.58	l
	Summer	20	17.5	22.5	20.00	2.50	a	39	41	38	39.33	1.53	m
	Autumn	20	17.5	15	17.50	2.50	ab	48	49	48	48.33	0.58	k
	Winter	15	17.5	15	15.83	1.44	bc	51	50	52	51.00	1.00	j
‘Sunshine’	Spring	15	10	12.5	12.50	2.50	d	76	77	77	76.67	0.58	e
	Summer	12.5	15	12.5	13.33	1.44	cd	72	75	73	73.33	1.53	f
	Autumn	12.5	12.5	10	11.67	1.44	de	79	80	78	79.00	1.00	d
	Winter	7.5	10	10	9.17	1.44	ef	81	84	82	82.33	1.53	c
‘Hongjixing’	Spring	7.5	5	5	5.83	1.44	gh	70	71	69	70.00	1.00	h
	Summer	7.5	7.5	10	8.33	1.44	fg	62	59	60	60.33	1.53	i
	Autumn	2.5	5	5	4.17	1.44	hi	72	71	70	71.00	1.00	gh
	Winter	2.5	2.5	0	1.67	1.44	ij	71	73	72	72.00	1.00	fg
‘Dolly’	Spring	2.5	0	0	0.83	1.44	j	89	90	88	89.00	1.00	a
	Summer	2.5	0	2.5	1.67	1.44	ij	87	89	85	87.00	2.00	b
	Autumn	0	0	0	0.00	0.00		0	0	0	0.00	0.00	
	Winter	0	0	0	0.00	0.00		0	0	0	0.00	0.00	

Table S3. Detailed data for the effect of low temperature (4 °C) on ovule culture in *Gerbera hybrida*.

Cultivar	Treatment time (Days)	Adventitious bud induction rate (%)						Adventitious bud induction time (Days)					
		Repeat 1	Repeat 2	Repeat 3	Average	SD	Sig. (5%)	Repeat 1	Repeat 2	Repeat 3	Average	SD	Sig. (5%)
'Prince'	0	20	22.5	20	20.83	1.44	b	39	38	39	38.67	0.58	gh
	3	22.5	25	20	22.50	2.50	ab	41	39	40	40.00	1.00	g
	7	25	27.5	25	25.83	1.44	a	37	38	37	37.33	0.58	hi
	10	27.5	22.5	22.5	24.17	2.89	ab	37	37	36	36.67	0.58	i
'Sunshine'	0	10	15	12.5	12.50	2.50	de	69	70	70	69.67	0.58	d
	3	12.5	15	17.5	15.00	2.50	cd	70	70	71	70.33	0.58	d
	7	17.5	20	15	17.50	2.50	c	72	73	73	72.67	0.58	c
	10	12.5	15	12.5	13.33	1.44	d	74	75	73	74.00	1.00	c
'Hongjixing'	0	7.5	7.5	10	8.33	1.44	f	62	64	63	63.00	1.00	e
	3	7.5	10	10	9.17	1.44	ef	63	61	63	62.33	1.15	e
	7	15	12.5	10	12.50	2.50	de	59	58	57	58.00	1.00	f
	10	7.5	10	10	9.17	1.44	ef	58	59	60	59.00	1.00	f
'Dolly'	0	0	0	0	0.00	0.00		0	0	0	0.00	0.00	
	3	0	2.5	2.5	1.67	1.44	g	85	85	86	85.33	0.58	b
	7	2.5	0	2.5	1.67	1.44	g	88	89	88	88.33	0.58	a
	10	0	0	0	0.00	0.00		0	0	0	0.00	0.00	

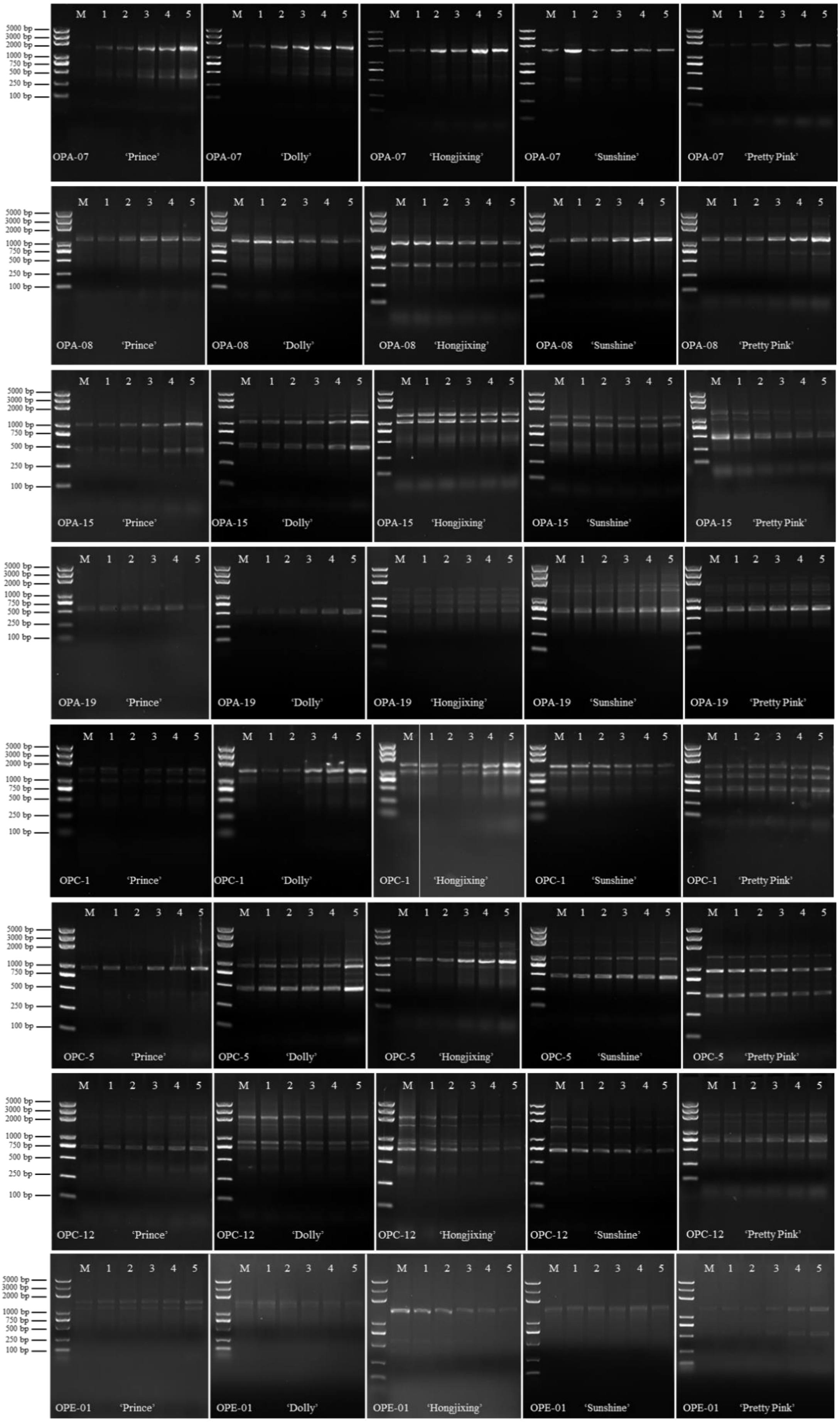

Figure S1. The amplified fragments generated by eight random amplified

polymorphic DNA (RAPD) primers in five *Gerbera hybrida* cultivars. The grouping of gels cropped from different gels, or from different parts of the same gel were separated by white dividing lines.
